# Development of a Deep Neural Network for Speeding Up a Model of Loudness for Time-Varying Sounds

**DOI:** 10.1177/2331216520943074

**Published:** 2020-08-27

**Authors:** Josef Schlittenlacher, Richard E. Turner, Brian C. J. Moore

**Affiliations:** 1 Department of Experimental Psychology, University of Cambridge; 2 Department of Engineering, University of Cambridge

**Keywords:** loudness model, instantaneous loudness, deep neural network, loudness meter

## Abstract

The “time-varying loudness” (TVL) model of Glasberg and Moore calculates “instantaneous loudness” every 1 ms, and this is used to generate predictions of short-term loudness, the loudness of a short segment of sound, such as a word in a sentence, and of long-term loudness, the loudness of a longer segment of sound, such as a whole sentence. The calculation of instantaneous loudness is computationally intensive and real-time implementation of the TVL model is difficult. To speed up the computation, a deep neural network (DNN) was trained to predict instantaneous loudness using a large database of speech sounds and artificial sounds (tones alone and tones in white or pink noise), with the predictions of the TVL model as a reference (providing the “correct” answer, specifically the loudness level in phons). A multilayer perceptron with three hidden layers was found to be sufficient, with more complex DNN architecture not yielding higher accuracy. After training, the deviations between the predictions of the TVL model and the predictions of the DNN were typically less than 0.5 phons, even for types of sounds that were not used for training (music, rain, animal sounds, and washing machine). The DNN calculates instantaneous loudness over 100 times more quickly than the TVL model. Possible applications of the DNN are discussed.

There are many practical applications of devices/methods for estimating the loudness of sounds in real time based on loudness models (Chalupper & Fastl, 2002; Glasberg & Moore, 2002; Zwicker, 1977), in other words of loudness meters (Fastl, 1993; Stone et al., 1997). For example, they may be used to control the loudness of commercials in broadcasting or to regulate the relative levels of the voices of different speakers in teleconferencing. However, loudness meters have involved compromises; in order to achieve real-time performance, the computational models of loudness on which they are based have to be simplified and approximations have to be made (Stone et al., 1997), which can lead to reduced accuracy. In this study, we describe a method for the rapid calculation of loudness using a deep neural network (DNN) that was trained using the predictions of a model for the loudness of time-varying sounds, the time-varying loudness (TVL) model of Glasberg and Moore (2002). The use of the DNN potentially allows real-time calculation of loudness with minimal reduction in accuracy.

To our knowledge, a preprint describing the present work (Schlittenlacher et al., 2019) was the first to use knowledge distillation via DNNs (Hinton et al., 2015) for perceptual models, but further work has been presented at conferences since then: see Van Den Broucke et al. (2020) for a transmission-line model of the cochlea, Sharma et al. (2019) for a speech-quality model, and Cox et al. (2020) for a glimpse-based speech-intelligibility model. DNNs have also been used for other tasks in the hearing domain, for example, to select hearing-aid fittings (Arntsen et al., 1996) and to separate speech from noise (see Wang & Chen, 2018, for an overview).

A block diagram of the TVL model is shown in Figure 1. The model includes a sequence of stages to simulate the transmission of sound to the eardrum (Shaw & Vaillancourt, 1985), the transmission of sound through the middle ear (Aibara et al., 2001), the frequency analysis that takes place in the cochlea (resulting in an auditory excitation pattern; Glasberg & Moore, 1990; Moore & Glasberg, 1983), the creation of a specific loudness pattern (including the effects of the compression that occurs in the cochlea; Moore & Oxenham, 1998), and summation of specific loudness across characteristic frequencies (Zwicker & Scharf, 1965) to give instantaneous loudness. Within the model, frequency is transformed to the ERB_N_-number scale, which has units Cams (Glasberg & Moore, 1990; Moore, 2012). This is a perceptually relevant scale comparable to a scale of distance along the basilar membrane (Moore, 1986). Instantaneous loudness is assumed to be an intervening variable, not available to conscious perception, although it has been shown that certain cortical regions show activity that is correlated with the instantaneous loudness calculated using the model (Thwaites et al., 2016). Instantaneous loudness represents a loudness scalar for a single point in time, based on the sound’s short-term spectrum at this time.

**Figure 1.**
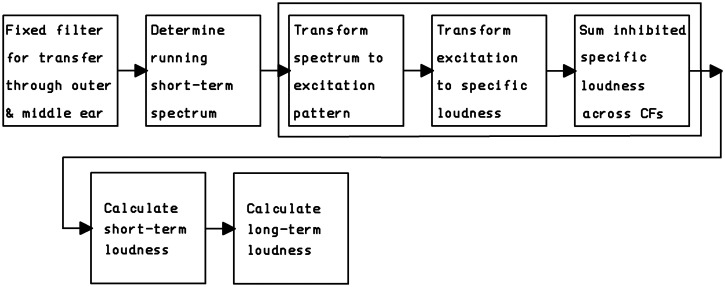
Block Diagram of the TVL Model of Glasberg and Moore (2002). The large rectangle outlines the stages replaced by the DNN. CFs = characteristic frequencies.

The instantaneous loudness is smoothed over time to calculate short-term loudness, which is meant to represent the loudness of a short piece of sound such as a single word in a sentence or a note in a piece of music. The short-term loudness is itself further smoothed over time to calculate the long-term loudness, which is meant to represent the overall loudness of a longer stretch of sound, such as a whole sentence or a musical phrase. The peak value of the long-term loudness gives good predictions of judged loudness for a variety of sounds, including amplitude compressed speech (Moore et al., 2003), sonic booms and impact sounds (Marshall & Davies, 2007), machinery sounds (Rennies et al., 2015), and speech processed to have increased or decreased dynamic range (Zorila et al., 2016). The model forms the basis of a proposed ISO standard (ISO 532-3, 2020), although the model used in the standard includes additional stages to account for the way that loudness is combined across ears (Moore & Glasberg, 2007; Moore et al., 2016).

Computationally, the most time-consuming stage of the TVL model is the calculation of the excitation pattern, which is estimated from the short-term spectrum of the sound and is used to calculate instantaneous loudness at 1-ms intervals. The excitation pattern is defined as the output of the auditory filters as a function of center frequency (Moore & Glasberg, 1983). It is estimated by calculating the outputs of an array of level-dependent auditory filters in response to each component of the input signal (after outer- and middle-ear filtering; Glasberg & Moore, 1990; Moore et al., 1997). The number of computations required to calculate instantaneous loudness makes it difficult to implement the TVL model in real time. To overcome this difficulty, we developed and trained a DNN to speed up the computation of instantaneous loudness from the momentary spectrum (third to fifth stages in Figure 1, outlined by the rectangular box), allowing real-time implementation. The DNN was trained to predict instantaneous loudness using a large database of speech sounds and artificial sounds (tones alone, bandpass filtered and notched noises, and tones in white or pink noise), with the predictions of the TVL model as a reference (providing the “correct” answer, specifically the loudness level in phons). Speech stimuli were selected for training because of the importance of speech for many applications and because speech has strong spectral and temporal fluctuations. The artificial sounds were included to improve the generalization and accuracy of the model for extreme cases, such as sounds with strong tonal components or with spectral notches. By avoiding the use of other categories for training, such as environmental sounds, we were able to assess how well the trained DNN generalized to unseen sounds.

## Structure and Training of the DNN

The stimuli used for training had a sampling rate of 16 kHz. Spectra were initially calculated using a 1,024-point discrete Fourier Transform, with successive windows being shifted by 560 samples. Then bins were grouped to form 61 bands with center frequencies up to 8 kHz, with one bin per band for center frequencies up to 0.2 kHz and 1/9th-octave wide bands for higher center frequencies. Sixty-one bands rather than the 512 bins of the Fourier Transform were chosen to reduce the number of weights in the DNN, permitting faster training. The 1/9th-octave wide bands still provided several inputs per ERB_N._ The limit of 8 kHz was chosen due to the sampling rate of the training material. The magnitude of the spectrum was expressed in decibels.

Both accuracy and computation speed were important considerations when choosing the structure of the DNN. The DNN was designed to replace the computationally most expensive part of the TVL model, the calculation of instantaneous loudness from the short-term spectrum, that is, the loudness for a single time frame (the third to fifth stages in Figure 1). The final DNN was a multilayer perceptron that consisted of an input layer with 61 units (corresponding to the 61 frequency bands), three hidden layers with 150 units each, and a single output unit with linear activation. The factors leading to this choice of structure are described here. The output of the DNN was a scalar representing the instantaneous loudness level in phon. This output format was chosen because of its similarity to the input scale. Both scales range roughly from 0 to 110, and the just noticeable diﬀerence in loudness is roughly constant on these scales. This facilitated the DNN in developing the mapping from input to output without the need for scale transformations. Simple “rectified linear unit” activations (Nair & Hinton, 2010) were used.

Alternative architectures were also considered. Convolutional neural networks (Fukushima, 1980), which are frequently used in image processing and applications where some of the latent features are invariant to shifts, did not achieve the same accuracy. This was probably because the input scale used (logarithmic frequency) did not allow the network to simulate filters that were valid over the whole range of the ERB_N_-number scale that is used in the TVL model. For example, the octave from 200 to 400 Hz spans 3.6 Cams but the octave from 4000 to 8000 Hz spans 6.2 Cams.

The DNN was optimized with regard to the root-mean-square (RMS) error from the predictions of the TVL model. The Adam gradient-descent optimizer (Kingma & Ba, 2014) was used with its default parameters. All weights of the DNN were initialized randomly. Three sets of data were used for training and choosing the hyperparameters (i.e., number of hidden units per layer and number of layers). First, 500,000 spectra were calculated from the LibriSpeech corpus (Panayotov et al., 2015), using the “clean” development set. These sounds were scaled so that each input file (typically a sentence) had an RMS level of 60 dB SPL. Second, about 700,000 pure tones with levels ranging from 15 to 110 dB SPL and various levels of background noise (from inaudible up to 10 dB below the level of the pure tone) were generated. Third, about 500,000 spectra of bandpass filtered noises and noises with spectral notches were generated. They had various overall levels, bandwidths, notch widths, and spectral gradients. To check for “over-fitting,” the performance of the DNN was assessed after training for 220, 1,000 and 5,000 epochs, where an epoch is a complete pass over the entire dataset one time.

The choice of 150 hidden units per layer was motivated by the resolution of specific loudness along the frequency axis in the TVL model; specific loudness is calculated for Cam values from 1.75 to 39 in 0.25-Cam steps. The data described earlier were split into a training set (90%) and a validation set (10%) after a random shuffle. The validation set was used for rough tuning of hyperparameters: 150 hidden units and three layers produced an RMS error of 0.3 phons for the validation set and gave more accurate predictions than deeper networks with up to nine layers and 75 hidden units (validation RMS error 0.5 phons). Architectures with 150 hidden units for each of four layers; 300 hidden units for each of three layers; or a narrowing structure of 600, 300, and 150 units for successive layers did not yield more accurate predictions than the chosen architecture. Predictions were less accurate when 150 hidden units and two layers were used (validation RMS error 0.7 phons).

## Assessment of the DNN

### Predictions of the Instantaneous Loudness of Speech and Everyday Sounds

**Figure 2.**
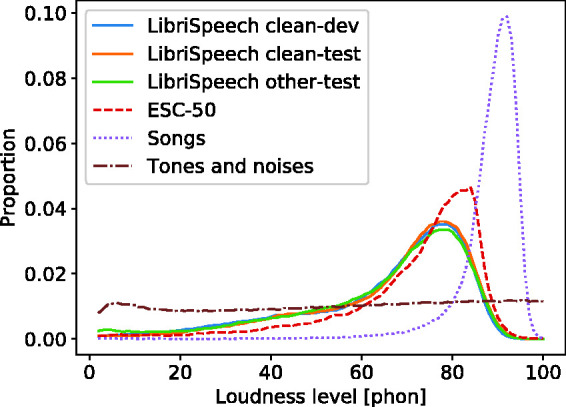
Distributions of Calculated Instantaneous Loudness Levels in the Training and Test Sets. The test sets were LibriSpeech (solid line), ESC-50 (dashed line), 1960s songs (dotted line), and artificial sounds (dash-dotted line).

Instantaneous loudness was predicted for two further sets of data from the LibriSpeech corpus: “clean” test set and “other” test set; neither of these sets of data was used for training. Each of them consisted of 500,000 spectra and they were scaled so that each file (typically a sentence) had an RMS level of 60 dB SPL. Loudness was also predicted for 250,000 spectra derived from the ESC-50 corpus (Piczak, 2015). This corpus contains 50 categories of environmental sounds, for example rain, animals, aircraft, keyboard typing, and washing machine. The sounds were again scaled to have an RMS level of 60 dB SPL. Finally, loudness was predicted for 100,000 spectra from 20 popular songs of the 1960s, which were scaled to have an RMS level of 70 dB SPL for each song. The slightly higher level was chosen because music is often listened to at a higher level than speech. The distributions of instantaneous loudness levels for all test and training sets as calculated by the TVL model are shown in Figure 2. Their modes are at loudness levels higher than the respective RMS sound levels, mainly due to spectral loudness summation (Zwicker et al., 1957). The distributions for speech and the environmental sounds extend to low loudness levels, corresponding, for example, to pauses between words and have standard deviations between 16 and 20 phons. The distribution for the songs is rather narrow, probably because they were amplitude compressed during the production process, and has a standard deviation of 7 phons.

Table 1 shows the RMS error in phons between the predictions of the TVL model and the predictions of the DNN after training for 220, 1,000, and 5,000 epochs. The errors did not vary systematically with the predicted loudness level, except for a small increase at very low levels, and the errors had a Gaussian distribution. After 1,000 epochs, the RMS error was below 0.5 phons for all classes of sounds. After 5,000 epochs, the RMS error increased slightly for the LibriSpeech “other” and ESC-50 sounds, which is a sign of “over-fitting.” Therefore, in what follows, we focus on the results achieved after training for 1,000 epochs.

**Table 1.** RMS Difference (Error) in Phons Between the Instantaneous Loudness Predicted by the TVL Model and by the DNN.

	Number of epochs		
Test material	220	1000	5000
Validation set	0.51	0.33	0.34
LibriSpeech “clean-test”	0.35	0.27	0.28
LibriSpeech “other-test”	0.55	0.45	0.47
ESC-50	0.56	0.45	0.47
Songs from the 1960s	0.38	0.35	0.31

The LibriSpeech “clean” sounds from the development set and a variety of tones and noises were used for training, while the remaining sounds were not used for training.

**Figure 3.**
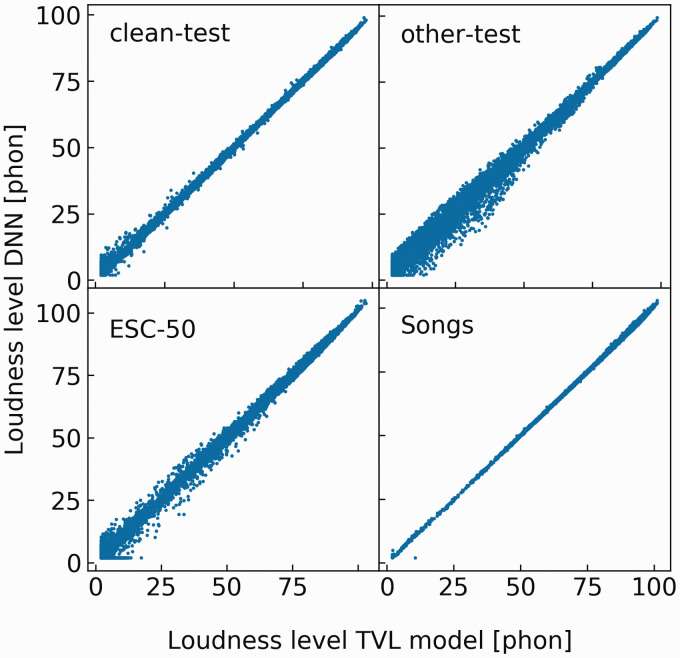
Scatter Plots Showing the Predictions of the DNN Versus the Instantaneous Loudness Level Calculated Using the TVL Model for the Four Test Sets. The four test sets include LibriSpeech “clean” test, LibriSpeech “other” test, 1960s songs and ESC-50, clockwise from upper left. Each panel shows more than 100,000 data points. DNN = deep neural network; TVL = time-varying loudness.

Table 2 shows various error measures for the DNN. The mean absolute error was 0.2 to 0.3 phons for each corpus, and smaller than 1.5 phons for 99% of all spectra for each corpus. The errors were not markedly lower for the two parts of the training set, the LibriSpeech “clean” development set and the artificial sounds (bottom two rows), than for the other sets, suggesting that the DNN generalizes well. Figure 3 shows scatter plots of instantaneous loudness values predicted by the TVL model (abscissa) and by the DNN (ordinate) for each of the four test sets. Note that the instantaneous loudness levels cover a wide range even for the stimuli whose overall RMS level was fixed. Outliers mostly occurred at low loudness levels, to which the DNN was exposed less during training.

**Table 2.** Error Measures of the Differences in Phons Between the Instantaneous Loudness Predicted by the TVL Model and by the DNN.

Test material	Root-mean-square error	Mean absolute error	99-percentile of absolute error	Maximum absolute error	Prediction bias
LibriSpeech “clean-test”	0.3	0.2	0.7	8.6	0.1
LibriSpeech “other-test”	0.4	0.2	1.3	15.7	0.1
ESC-50	0.4	0.3	1.4	15.3	0.2
Songs from the 1960s	0.4	0.3	0.9	10.3	0.3
LibriSpeech “clean-dev”	0.3	0.2	0.7	7.5	0.1
Tones and noises	0.4	0.3	1.1	6.8	0.1

The LibriSpeech “clean” sounds from the development set and tones and noises were used for training. The last two rows show predictions for part of the training material (training and validation set collapsed).

To investigate the effect of the training material, we trained DNNs with the same structure but with different sets of data, similar to a cross validation. Four sets were used for training: the ESC-50 set, the 1960s songs, the LibriSpeech “clean” development set as a subset of the original training set, and the artificial sounds of the original training set. Table 3 shows the RMS errors in phons of predictions made by the DNNs trained on these four sets, with rows indicating the training set and columns indicating the test set. For each DNN, the training and validation RMS error were the same to one decimal place. The DNNs trained with speech or with the environmental sounds gave accurate predictions of the loudness of those same sounds and of the songs, even slightly better than the original DNN. However, they failed to predict the loudness of the artificial sounds, with RMS errors above 20 phons. Training with the 1960s songs, which had only a narrow range of loudness levels, led to consistently less accurate predictions. The artificial sounds produced moderately accurate predictions for all test sets, despite the fact that this set did not contain any real-world sounds. However, the RMS error was about 5 to 10 times that of the original DNN, being between 1.7 and 3.5 phons.

**Table 3.** Root-Mean-Square Errors in Phons of Predictions of DNNs With the Same Architecture as the Selected DNN but Trained on the Material Given by the Rows, and Evaluated Using the Material Given by the Columns.

	Evaluation material					
	LibriSpeech “clean-dev”	ESC-50	1960s songs	Tones and noises	LibriSpeech “clean-test”	LibriSpeech “other-test”
*Training material*					
LibriSpeech “clean-dev”	0.2	0.3	0.4	22.9	0.2	0.3
ESC-50	0.3	0.2	0.1	23.3	0.2	0.3
1960s songs	2.5	2.4	0.2	38.7	1.8	3.1
Tones and noises	3.3	3.0	1.7	0.4	3.4	3.5
A-weighted level	5.0	5.0	2.7	6.7	4.8	5.3

When the training and test sets were the same, the training RMS error and validation RMS error were the same within one decimal place. The last row shows RMS errors of loudness predictions using A-weighted sound pressure level, after correction for the prediction bias associated with the respective test set.

Because of the computational cost of loudness models, A-weighted SPL is frequently used as a proxy for loudness. The last row of Table 3 shows the RMS error when taking the A-weighted SPL as an estimator of the instantaneous loudness level predicted by the TVL model. The prediction bias of the test sets, that is, the mean difference between A-weighted SPL and loudness level of the TVL model, was subtracted from the A-weighted SPLs before calculating the RMS errors. The RMS errors were between 2.7 phons for the songs and 6.7 phons for the artificial sounds.

In summary, the original DNN, which was trained with both speech and artificial sounds, gave more accurate predictions than DNNs with the same architecture but trained with more restricted sets of materials, and also gave more accurate predictions than A-weighted SPL.

### Predictions of Short-Term, Long-Term, and Overall Loudness

As described earlier, the TVL model calculates short-term and long-term loudness from instantaneous loudness (Figure 1). To compare the predictions of the DNN with those of the TVL model for short-term and long-term loudness, instantaneous loudness was predicted once per 1 ms for the 2,620 sentences of the LibriSpeech “clean” test set and for the 2,000 five-second long sounds of ESC-50, with both the DNN and the TVL model. Short-term and long-term loudness were calculated using the formula of the TVL model. The maximum value of the long-term loudness was taken to represent the overall loudness (Zorila et al., 2016). The predicted values for overall loudness had a narrower distribution than for instantaneous loudness (Figure 2), as expected. The standard deviations for overall loudness were 2.3 phons for the LibriSpeech “clean” test set and 4.8 phons for the ESC-50 set, respectively, and the ranges were 15.9 phons (from 76.5 to 92.4) and 63.2 phons (from 39.9 to 103.0), respectively. Tables 4 and 5 show various error measures for the speech sounds and environmental sounds, respectively. The errors were somewhat smaller than those for instantaneous loudness. RMS errors were smaller than 0.4 phons and 99% of all loudness values based on the DNN were within 1.1 phons of the predictions of the TVL model. The prediction bias, which is the difference between the mean of the loudness levels predicted by the DNN and by the TVL model, was close to zero for all loudness metrics, implying that there was no systematic error.

**Table 4.** Various Measures of the Difference in Phons (error) Between the Predictions of the TVL Model and the DNN for the LibriSpeech “Clean-Test” Set for Short-Term, Long-Term, and Overall Loudness. Error Measures for Stationary Loudness are also shown.

	RMS error	Mean absolute error	99th-percentile of absolute error	Maximum absolute error	Prediction bias
Short-term loudness	0.2	0.2	0.4	8.3	−0.1
Long-term loudness	0.2	0.2	0.4	8.3	−0.2
Overall loudness	0.2	0.2	0.4	0.7	−0.2
Stationary loudness	1.6	1.2	3.9	6.3	0.6

The last row shows differences between the overall loudness predicted by the TVL model and stationary loudness (ISO 532-2, 2017), based on the average spectrum.

**Table 5.** Various Measures of the Difference in Phons (errors) Between the Predictions of the TVL Model and the DNN for the ESC-50 Set for Short-Term, Long-Term, and Overall Loudness. Error Measures for Stationary Loudness are also shown.

	RMS error	Mean absolute error	99th-percentile of absolute error	Maximum absolute error	Prediction bias
Short-term loudness	0.3	0.2	1.1	12.5	−0.2
Long-term loudness	0.3	0.3	0.9	12.4	−0.2
Overall loudness	0.4	0.4	1.1	3.6	−0.3
Stationary loudness	3.6	2.7	10.0	20.1	1.4

For comparison to another method that calculates overall loudness with a low computational cost, the average spectrum of each sound was taken and its loudness was calculated using ISO 532-2 (2017). For stationary diotic sounds, ISO 532-2 produces almost the same loudness values as the TVL model. For the speech sounds and environmental sounds, the RMS errors were 1.6 and 3.6 phons, respectively. For 1% of the sounds, the absolute errors were larger than 3.9 and 10.0 phons, respectively. Thus, overall loudness was predicted more accurately using the DNN than using the long-term average spectrum and ISO 532-2.

### Predictions for Pure Tones

**Figure 4.**
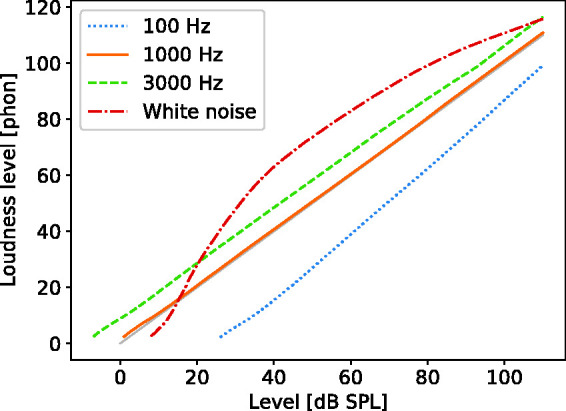
Loudness Level in Phons Predicted by the DNN as a Function of Sound Level for Pure Tones With Frequencies of 100, 1000, and 3000 Hz and for White Noise (15 to 8000 Hz).

Figure 4 shows loudness levels predicted by the DNN for pure tones in quiet as a function of input sound level for frequencies of 100 Hz (dotted line), 1000 Hz (solid line), and 3000 Hz (dashed line), assuming free-field presentation with frontal incidence. Note that pure tones with a wide range of levels and frequencies were included in the sounds used during training, so we expected these predictions to be accurate. The predictions are shown here to demonstrate that the inclusion of speech and noise bands in the training material did not adversely affect the accuracy of the predictions for the pure tones.

The predictions are consistent with empirical data (Hellman, 1976) and are almost identical to the predictions of the TVL model. For the 1000-Hz tone, by definition its loudness level in phons is equal to its physical level in dB SPL. The predictions of the DNN show this relationship almost exactly. The loudness level is greater for the 3000-Hz than for the 1000-Hz tone because 3000 Hz is close to the resonant frequency of the ear canal, so the sound level at the eardrum is boosted relative to that in free field (Shaw & Vaillancourt, 1985). The loudness level is lower at 100 Hz than at 1000 Hz partly because of the attenuation characteristic of the middle ear and partly because less gain is applied by the active mechanism in the cochlea at low frequencies (Cooper, 2004; Moore et al., 1997). Both of these effects are simulated in the TVL model.

### Predictions for Noises as a Function of Bandwidth

**Figure 5.**
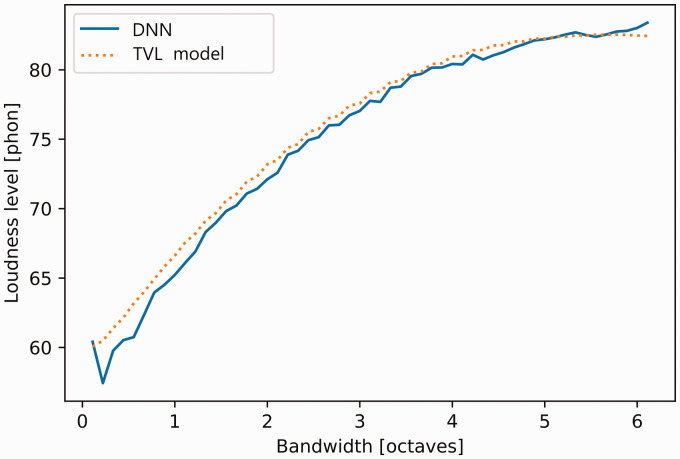
Loudness Level in Phons Predicted by the DNN (Solid Line) and by the TVL Model (Dashed Line) as a Function of the Bandwidth of a Pink Noise Geometrically Centered at 1 kHz with an Overall Level of 60 dB SPL. DNN = deep neural network; TVL = time-varying loudness.

Figure 5 shows the loudness level of bandpass filtered pink noise geometrically centered at 1 kHz, plotted as a function of bandwidth, as predicted by the TVL model and by the DNN. The overall level of the noise was 60 dB SPL. Again, it should be noted that noise bands with a wide range of levels, center frequencies, and bandwidths were included in the sounds used during training. The point here was to check that the inclusion of speech and pure tones in the training material did not affect the accuracy of the predictions for noise bands. For small bandwidths, the loudness level predicted by the DNN was slightly below that predicted by the TVL model. Overall, the predictions of the DNN for the loudness of bands of noise showed good accuracy.

The loudness level of white noise as a function of level as predicted by the DNN is shown in Figure 4 (dash-dotted line). Its threshold, corresponding to a loudness level of about 2 phons (Moore et al., 1997), is higher than for the 1-kHz and 3-kHz pure tones because for broadband noise the level at the output of any single auditory filter is much lower than the overall level. At medium levels, the loudness level of the white noise is considerably higher than for pure tones of the same level, because of spectral loudness summation, and this effect deceases at high levels, consistent with experimental data (Zwicker et al., 1957).

## Discussion

The predictions of the DNN for the environmental sounds and music were remarkably accurate. This is noteworthy, since the DNN was trained only using speech and synthetic sounds. This suggests that the DNN generalizes well to real-world sounds and would do so for sounds other than those tested here. The predictions for music were accurate despite the fact that the music test sounds were scaled to have an RMS level of 70 dB SPL, which is higher than the level of 60 dB SPL that was used with the speech sounds used for training. This shows that the DNN works well for sounds with levels that it was not exposed to frequently during training. However, there were some prediction errors for low loudness levels (Figure 3), to which the DNN was not exposed frequently during training (Figure 2).

Predictions were accurate for real-world sounds other than those used for training when the artificial sounds were not included in the training set (see Table 3). However, the catastrophic performance for pure tones and notched noises when the DNN was trained with real-world sounds indicates that the DNN can produce large errors for test materials that are very different from any training material. This may have occurred because when the artificial sounds were not used for training, the DNNs did not simulate the structure of the TVL model but instead performed a nonlinear regression in the 61-dimensional input space. For this reason, it was important to include the artificial sounds in the training set. The overall performance of the DNN might have been even better if the training sounds had included more sounds with lower levels. It might also be possible to achieve even better generalization by using an adversarial approach (Szegedy et al., 2013), in which a second DNN tries to find sounds for which the predictions of the first DNN are inaccurate, with the first DNN then adapting in order to achieve more accurate predictions for the problematic sounds. We leave this for a future study.

The predictions of short-term, long-term, and overall loudness based on the DNN were slightly more accurate than those for instantaneous loudness. One might have expected markedly smaller prediction errors because of the temporal smoothing involved. However, successive spectra in a sound are not independent, and this limits the improvement that can be expected. Both for instantaneous loudness and for overall loudness, the predictions of the DNN were much more accurate than those of other computationally cheap methods, specifically A-weighted SPL and stationary loudness calculated using ISO 532-2 (2017), which are frequently used in practical applications. The improvement for the DNN was a reduction of the RMS error in phons by a factor of 5 to 20, despite the fact that predictions based on A-weighted SPL were corrected for the prediction bias of the test set.

In its reference implementation, the TVL model needs about 50 ms to calculate a single instantaneous loudness value on a modern central processing unit (CPU; Intel i7 6th generation), although it is somewhat faster for simple input spectra like a pure tone without background noise. For applications where delays need to be kept small, it is important to perform a single instantaneous loudness calculation in real time, that is, faster than 1 ms. To do this, we implemented the trained DNN in Matlab. The loudness prediction for a single input spectrum took 0.3 ms when using a single CPU. Dedicated DNN hardware would be able to perform the computation even faster. When delays are allowed, for example when analyzing the loudness of long recordings, loudness calculations can be done in parallel. To calculate the 86,400,000 instantaneous loudness values of a 24-hr-long recording, our implementation of the DNN in TensorFlow/Python needed about 1 min on a graphic processor unit (Nvidia GeForce GTX 1080), and a few minutes on a CPU.

The present approach has some limitations. First, the frequency range had an upper limit of 8 kHz, due to the sample rate of the training material. This may be enough for many applications but is an octave lower than for the TVL model. Second, only a few low loudness levels of real-world sounds were included in the training material, which led to some large errors at these levels (see Figure 3 and the maximum absolute errors in Tables 2, 4, and 5). These limitations may be overcome by using training sets that have a higher sample rate and have a more balanced range of instantaneous loudness levels. For the present study, we intentionally chose speech as the only real-world sounds in the training set to investigate how well the DNN generalizes to completely unseen types of sound.

Potential applications of the DNN include development of a real-time loudness meter without the compromises that were necessary previously to achieve real-time operation (Stone et al., 1997) and real-time control of levels in broadcasting to ensure (among other things) that the advertisements are not louder than the main program material (Moore et al., 2003). The DNN could be extended to predict loudness for people with hearing loss (Moore & Glasberg, 1997, 2004). In principle, this could be used for on-line control of loudness in hearing aids so as to restore loudness perception more nearly to normal (Launer & Moore, 2003).

The extension to hearing loss could be done in two ways. Including parameters characterizing hearing impairment as part of the input, such as the proportion of hearing loss due to inner versus outer hair cell dysfunction at different frequencies (Moore & Glasberg, 1997, 2004), would require a considerably larger amount of training data to cover all possible sorts of hearing loss, and it is difficult to predict how well a DNN trained in this way would generalize to unseen hearing losses. Another approach would be to use a loudness model for impaired hearing (Moore & Glasberg, 2004) to generate the “correct” loudness values for a specific hearing loss during the training of the DNN. In this case, the trained DNN would only be valid for that specific hearing loss. However, the “correct” loudness calculations only need to be done once during training, and thus, this approach is suitable for application in a hearing aid using loudness predicted via a DNN. Furthermore, it can be anticipated that a DNN trained on a specific hearing loss has about the same performance as a DNN trained for normal hearing—the input space remains the same and normal hearing is a special case in the hearing-loss loudness model.

## Conclusions

The DNN gave accurate predictions of loudness for environmental sounds and music despite training using speech and synthetic sounds only. This shows good generalization and suggests that the DNN will give reasonably accurate predictions for a wide variety of everyday sounds. Most predictions were accurate, with RMS errors of 0.5 phons or less, a difference in loudness level that would not be detectable. This was about 5 to 20 times better than metrics that are frequently used in practice, such as A-weighted SPL and loudness calculated from the long-term average spectrum. The DNN calculates instantaneous loudness more than 100 times faster than the TVL model, making real-time implementation possible. This opens up potential applications in broadcasting and in the on-line control of loudness in hearing aids.
